# Supplementary material for: A Likelihood Approach for Real-Time Calibration of Stochastic Compartmental Epidemic Models
Source: PLoS Comput Biol. 2017 Jan 17;13(1):e1005257. doi: 10.1371/journal.pcbi.1005257 (PMC5240920; doi:10.1371/journal.pcbi.1005257)
Supplement: S1 File — (TAR.GZ) [file pcbi.1005257.s014.tar.gz › HSPH_Online-SI-Revision/IPoi09/n1-mild/n1-mild_BENCH_table4.pdf]

1	R0	{1.27677, 7.89375}	{2.05892, 16.899}	{{1.92096, 15.109}, {2.2194, 19.1478}}	{61.2603, 114.08}	inf	18.	111.945	{56., 157.}	521.919	0.76194	newly	14.	42.8545	{19., 64.}	207.403	0.451731	Reff	0.891185	0.669006	{0.453832, 0.989824}	33.4337	ar	400.	817.055	{773., 853.}	104.264
2	R0	{1.33231, 10.5259}	{2.1964, 15.5912}	{{2.04423, 14.1631}, {2.35866, 16.9062}}	{64.8567, 48.1218}	inf	9.	25.7424	{7., 66.}	199.832	0.386456	newly	6.	10.1364	{2., 23.}	104.293	0.321648	Reff	0.735435	0.394779	{0.312014, 0.479029}	46.3203	ar	467.	837.485	{804., 870.}	79.3329
3	R0	{1.2273, 6.76944}	{1.54686, 5.31873}	{{1.54465, 5.30296}, {1.54465, 5.30296}}	{26.0375, 21.5833}	inf	10.	7.25	{0., 18.}	73.9286	0.708259	newly	13.	8.	{0., 18.}	68.1319	0.76145	Reff	0.924157	0.608825	{0.455672, 0.758423}	34.1211	ar	314.	620.	{555., 705.}	97.4522
4	R0	{1.41215, 17.5742}	{1.54173, 10.453}	{{1.48482, 9.52864}, {1.57414, 10.8293}}	{9.17572, 40.5207}	inf	22.	22.05	{6., 43.}	53.6364	0.270603	newly	12.	13.275	{2., 28.}	63.5417	0.297816	Reff	1.14808	0.666841	{0.527337, 0.864877}	41.9168	ar	465.	616.85	{541., 683.}	32.6559
5	R0	{1.42222, 6.15886}	{2.27132, 14.9385}	{{2.11292, 14.2212}, {2.42074, 16.0321}}	{59.7025, 142.554}	inf	19.	85.0952	{41., 131.}	347.87	0.600176	newly	13.	34.2698	{17., 56.}	165.568	0.369494	Reff	0.797865	0.484525	{0.335958, 0.60189}	43.0882	ar	490.	853.143	{808., 888.}	74.1108
6	R0	{1.24363, 9.95596}	{2.09126, 14.6853}	{{1.98025, 11.3733}, {2.29738, 18.743}}	{68.158, 47.5024}	inf	38.	148.982	{85., 210.}	292.058	0.572561	newly	28.	69.	{43., 88.}	146.429	0.37868	Reff	0.93521	0.981058	{0.648808, 1.39801}	24.3102	ar	411.	815.321	{759., 884.}	98.375
7	R0	{1.36478, 5.96056}	{2.09109, 12.1864}	{{1.98131, 11.1772}, {2.22518, 13.5548}}	{53.2179, 104.45}	inf	20.	79.322	{32., 162.}	297.966	0.52582	newly	19.	40.1525	{16., 79.}	121.142	0.303942	Reff	0.848893	0.576373	{0.392299, 0.73876}	41.4218	ar	484.	818.983	{767., 857.}	69.2114
8	R0	{1.36335, 17.6429}	{1.76066, 19.312}	{{1.73196, 18.9248}, {1.79789, 19.6311}}	{29.1419, 9.46031}	inf	41.	81.0612	{43., 114.}	103.584	0.301003	newly	19.	27.6122	{14., 42.}	58.8614	0.207822	Reff	1.08932	0.908066	{0.6048, 1.32042}	26.7331	ar	484.	707.551	{662., 768.}	46.1882
9	R0	{1.26575, 5.28707}	{2.01654, 19.3966}	{{1.98971, 18.8819}, {2.11476, 19.6444}}	{59.316, 266.869}	inf	24.	94.6909	{54., 140.}	294.545	0.567465	newly	28.	33.0909	{17., 53.}	37.6623	0.147475	Reff	0.731603	0.671553	{0.427302, 1.0247}	27.469	ar	472.	794.818	{748., 837.}	68.3937
10	R0	{1.42942, 9.49905}	{2.26926, 19.3353}	{{2.26161, 19.0355}, {2.29738, 19.5128}}	{58.754, 103.55}	inf	17.	60.806	{24., 118.}	258.385	0.491017	newly	11.	19.4925	{6., 39.}	93.4871	0.258159	Reff	0.773316	0.411799	{0.296271, 0.522432}	47.3114	ar	468.	859.552	{828., 891.}	83.665
11	R0	{1.46827, 15.0983}	{1.75874, 19.3343}	{{1.73196, 19.1609}, {1.79789, 19.6311}}	{19.7831, 28.056}	inf	18.	87.8696	{44., 121.}	416.88	0.68772	newly	6.	31.3913	{19., 43.}	423.188	0.691763	Reff	1.17902	0.953749	{0.63653, 1.39417}	26.8106	ar	495.	714.978	{667., 773.}	44.4401
12	R0	{1.25751, 13.279}	{1.74677, 19.2139}	{{1.73196, 18.9248}, {1.75837, 19.4054}}	{38.9072, 44.6939}	inf	33.	84.4773	{45., 114.}	159.711	0.40134	newly	20.	29.5	{15., 43.}	59.3182	0.208302	Reff	1.01481	0.942675	{0.646164, 1.35027}	23.2686	ar	366.	703.75	{644., 766.}	92.2814
13	R0	{1.3425, 9.16361}	{2.05152, 13.8137}	{{1.93231, 12.4507}, {2.16755, 14.8261}}	{52.813, 50.7456}	inf	14.	29.4386	{9., 57.}	130.576	0.314758	newly	8.	13.7719	{3., 27.}	105.482	0.354674	Reff	0.765225	0.460656	{0.348976, 0.583426}	40.1512	ar	442.	803.14	{748., 847.}	81.706
14	R0	{1.18936, 6.20583}	{2.03759, 12.69}	{{1.94282, 12.0157}, {2.14401, 13.8218}}	{71.3181, 104.485}	inf	14.	65.2632	{26., 119.}	368.17	0.606611	newly	10.	31.5789	{10., 53.}	220.702	0.484169	Reff	0.752865	0.536829	{0.412019, 0.693587}	30.6148	ar	382.	802.719	{764., 842.}	110.136
15	R0	{1.27137, 9.78868}	{1.93297, 12.7752}	{{1.83592, 11.3493}, {2.01705, 14.0718}}	{52.0383, 30.5103}	inf	28.	72.0638	{30., 122.}	160.106	0.373437	newly	19.	35.9149	{17., 59.}	96.8645	0.264761	Reff	0.827662	0.615705	{0.436927, 0.805969}	31.5497	ar	496.	779.34	{723., 823.}	57.1251
16	R0	{1.23102, 13.6452}	{1.66395, 10.8897}	{{1.63746, 10.1332}, {1.67361, 11.759}}	{35.1687, 20.1943}	inf	35.	37.3	{8., 76.}	60.8571	0.305481	newly	16.	21.12	{6., 44.}	72.75	0.287858	Reff	0.923265	0.632986	{0.480643, 0.801891}	31.4404	ar	445.	676.2	{621., 732.}	51.9551
17	R0	{1.27213, 13.7452}	{1.69159, 12.543}	{{1.58389, 10.7075}, {1.77361, 14.2286}}	{32.9728, 12.2587}	inf	29.	61.6	{24., 102.}	123.824	0.352587	newly	12.	31.9091	{11., 54.}	175.	0.429025	Reff	1.02661	0.807554	{0.577444, 1.1035}	27.5238	ar	367.	660.036	{594., 746.}	82.9279
18	R0	{1.25318, 11.2151}	{1.83933, 18.7057}	{{1.79724, 16.8903}, {1.87294, 19.1994}}	{46.773, 66.7901}	inf	28.	93.4259	{46., 132.}	234.193	0.496546	newly	18.	33.8889	{19., 48.}	93.8272	0.277743	Reff	0.917328	0.818817	{0.560009, 1.1259}	23.4527	ar	416.	747.093	{701., 795.}	79.5896
19	R0	{1.46006, 8.11496}	{2.15891, 12.7957}	{{2.04871, 11.6322}, {2.30907, 14.464}}	{47.8642, 57.6809}	inf	28.	63.8871	{22., 109.}	136.924	0.334969	newly	24.	30.6774	{13., 51.}	54.3011	0.20384	Reff	0.861435	0.489881	{0.354792, 0.628335}	46.8584	ar	496.	834.968	{798., 873.}	68.3403
20	R0	{1.14273, 6.71261}	{2.00598, 16.5715}	{{1.92096, 15.109}, {2.12588, 17.7287}}	{75.5431, 146.871}	inf	18.	102.034	{56., 151.}	466.858	0.723949	newly	18.	40.0172	{20., 60.}	122.51	0.314847	Reff	0.787341	0.693146	{0.444963, 0.990065}	27.7707	ar	362.	798.466	{751., 843.}	120.571
21	R0	{1.42432, 8.23905}	{1.82536, 11.026}	{{1.75469, 10.2835}, {1.83592, 11.1369}}	{28.1566, 33.8256}	inf	21.	22.4565	{6., 49.}	61.3872	0.281636	newly	16.	12.6957	{2., 26.}	55.163	0.357021	Reff	0.914413	0.522951	{0.433277, 0.626049}	42.8102	ar	405.	738.043	{701., 784.}	82.233
22	R0	{1.29386, 17.863}	{1.62743, 12.4691}	{{1.53473, 10.5556}, {1.70173, 14.7029}}	{25.7809, 30.1962}	inf	28.	60.0385	{27., 94.}	120.33	0.317982	newly	12.	31.4615	{15., 46.}	166.026	0.394581	Reff	1.06614	0.820986	{0.633843, 1.1085}	26.8313	ar	488.	638.712	{555., 715.}	30.8835
23	R0	{1.39274, 13.0167}	{1.89696, 15.9098}	{{1.88811, 15.9062}, {1.8951, 16.0085}}	{36.2036, 22.2261}	inf	35.	30.907	{11., 62.}	60.598	0.311956	newly	27.	12.3488	{4., 25.}	60.2929	0.47284	Reff	0.891354	0.505881	{0.365754, 0.624964}	43.2457	ar	475.	766.86	{714., 815.}	61.4443
24	R0	{1.32178, 15.8991}	{2.01056, 19.1492}	{{1.98971, 18.8819}, {2.11476, 19.6444}}	{52.1103, 20.4745}	inf	48.	109.262	{71., 150.}	128.996	0.343247	newly	20.	38.	{20., 56.}	94.2623	0.270319	Reff	0.896167	0.802362	{0.509416, 1.25551}	31.0874	ar	471.	798.033	{754., 845.}	69.4337
25	R0	{1.37568, 17.8427}	{1.638, 14.0304}	{{1.58973, 12.6816}, {1.67042, 15.6632}}	{19.0682, 21.3661}	inf	27.	47.3673	{17., 90.}	90.8541	0.260261	newly	11.	23.0408	{9., 40.}	119.109	0.307367	Reff	1.05377	0.759837	{0.545416, 1.11842}	30.7692	ar	398.	652.347	{549., 721.}	63.9063
26	R0	{1.20993, 6.4656}	{1.94492, 15.5202}	{{1.87975, 14.1819}, {2.00049, 16.7932}}	{60.7644, 140.042}	inf	9.	76.1525	{36., 122.}	746.139	0.881704	newly	13.	32.7797	{14., 56.}	154.498	0.365384	Reff	0.834852	0.676515	{0.44811, 1.10624}	33.7046	ar	345.	778.017	{726., 829.}	125.512
27	R0	{1.20548, 13.8003}	{1.86544, 19.0882}	{{1.8189, 18.8228}, {1.87294, 19.9256}}	{54.7466, 38.3173}	inf	30.	91.2545	{47., 127.}	204.667	0.457044	newly	10.	32.5636	{17., 47.}	225.636	0.481376	Reff	0.876384	0.786379	{0.54128, 1.20229}	25.0798	ar	350.	749.764	{692., 803.}	114.218
28	R0	{1.3176, 14.6594}	{1.78311, 17.3638}	{{1.64814, 14.7029}, {1.87294, 19.3194}}	{35.3304, 19.5103}	inf	22.	89.46	{33., 137.}	314.818	0.604384	newly	16.	36.76	{16., 53.}	140.75	0.415859	Reff	1.11732	1.13935	{0.80173, 1.56899}	21.5079	ar	362.	712.32	{654., 765.}	96.7735
29	R0	{1.28202, 6.23047}	{2.13413, 18.2775}	{{1.98261, 16.2766}, {2.26107, 19.9579}}	{66.4661, 193.357}	inf	11.	88.2321	{46., 143.}	702.11	0.859712	newly	14.	32.5893	{13., 55.}	137.117	0.331549	Reff	0.844851	0.55977	{0.374092, 0.721995}	40.0652	ar	391.	827.179	{777., 866.}	111.555
30	R0	{1.28177, 8.83252}	{1.94556, 11.8619}	{{1.86868, 10.9611}, {2.04669, 13.0262}}	{51.7866, 34.2979}	inf	22.	54.537	{19., 98.}	154.798	0.362217	newly	11.	28.3889	{12., 50.}	164.478	0.373989	Reff	0.848532	0.528961	{0.392972, 0.670273}	37.7238	ar	427.	779.593	{729., 827.}	82.5744
31	R0	{1.35886, 5.97708}	{2.54596, 18.907}	{{2.36153, 17.2664}, {2.66225, 19.9254}}	{87.36, 216.325}	inf	14.	118.842	{64., 199.}	748.872	0.88764	newly	13.	40.6316	{21., 70.}	214.17	0.456932	Reff	0.712043	0.461886	{0.293929, 0.671507}	41.9087	ar	491.	896.105	{863., 923.}	82.5062
32	R0	{1.27601, 17.9804}	{1.74465, 17.4525}	{{1.66003, 15.2961}, {1.8189, 19.688}}	{36.7273, 10.3564}	inf	32.	73.381	{33., 121.}	136.31	0.390062	newly	12.	28.3333	{12., 46.}	144.444	0.398813	Reff	1.03995	0.878599	{0.614573, 1.38057}	29.4352	ar	445.	690.69	{626., 758.}	59.6843
33	R0	{1.3368, 11.3235}	{2.20724, 19.2509}	{{2.12588, 18.765}, {2.26161, 19.9579}}	{65.1136, 70.008}	inf	41.	127.957	{76., 169.}	212.091	0.476632	newly	18.	44.1857	{26., 60.}	145.476	0.367361	Reff	0.838174	0.69821	{0.440909, 1.02279}	30.6628	ar	417.	835.143	{796., 875.}	100.274
34	R0	{1.21021, 11.3635}	{1.5436, 9.86102}	{{1.51295, 9.08517}, {1.58389, 10.8293}}	{27.5483, 13.4076}	inf	19.	16.5102	{1., 37.}	60.58	0.458959	newly	11.	10.4286	{1., 22.}	65.3061	0.46658	Reff	0.958486	0.654004	{0.547435, 0.855849}	32.0021	ar	327.	611.041	{526., 683.}	86.8626
35	R0	{1.53382, 10.8866}	{2.25881, 19.2744}	{{2.2194, 19.0355}, {2.29738, 19.5128}}	{47.2672, 77.0473}	inf	49.	149.612	{91., 193.}	205.33	0.469451	newly	34.	52.5821	{30., 72.}	59.5698	0.195306	Reff	0.963239	0.714076	{0.4772, 1.07767}	33.2115	ar	476.	850.075	{810., 881.}	78.5871
36	R0	{1.20423, 6.83844}	{2.2849, 14.4317}	{{2.15363, 13.2892}, {2.42687, 16.0321}}	{89.7393, 111.038}	inf	19.	78.0156	{42., 123.}	310.609	0.573025	newly	18.	33.4844	{18., 52.}	92.2743	0.261133	Reff	0.714108	0.440876	{0.333135, 0.580613}	41.142	ar	416.	859.5	{830., 891.}	106.611
37	R0	{1.43629, 10.1548}	{1.88192, 15.9215}	{{1.85253, 15.8132}, {1.8951, 16.0085}}	{31.0266, 56.788}	inf	18.	57.1207	{22., 95.}	220.785	0.448778	newly	19.	22.2586	{8., 42.}	56.7151	0.232863	Reff	1.01977	0.61457	{0.447482, 0.831786}	42.3117	ar	367.	758.172	{697., 799.}	106.586
38	R0	{1.37504, 8.93428}	{2.39391, 15.7531}	{{2.28144, 14.5901}, {2.53519, 17.5902}}	{74.0971, 76.3221}	inf	26.	89.3714	{34., 142.}	244.286	0.469533	newly	22.	34.4286	{12., 56.}	75.7143	0.2357	Reff	0.764522	0.444945	{0.310001, 0.57527}	46.3542	ar	483.	875.143	{838., 910.}	81.189
39	R0	{1.51637, 9.36742}	{1.93741, 11.5476}	{{1.87584, 10.9611}, {1.99822, 12.2033}}	{27.766, 23.2738}	inf	18.	30.434	{9., 59.}	93.8155	0.285181	newly	14.	15.566	{4., 31.}	64.0162	0.28294	Reff	0.938633	0.488843	{0.395191, 0.618169}	48.2809	ar	441.	780.66	{732., 818.}	77.0205
40	R0	{1.23658, 9.82869}	{2.17897, 13.3586}	{{2.0968, 12.1899}, {2.28604, 14.5978}}	{76.2091, 35.9146}	inf	25.	72.9	{36., 133.}	193.067	0.416937	newly	12.	33.9	{14., 60.}	184.722	0.401337	Reff	0.746894	0.493787	{0.347016, 0.579989}	41.1046	ar	414.	839.55	{790., 872.}	102.79
41	R0	{1.24247, 5.4242}	{1.87629, 9.24452}	{{1.82266, 8.71672}, {1.93816, 9.82151}}	{51.0128, 70.431}	inf	10.	50.2759	{15., 97.}	402.07	0.612231	newly	10.	32.9138	{10., 69.}	233.966	0.440993	Reff	0.861032	0.597812	{0.437177, 0.728748}	35.385	ar	345.	751.017	{711., 798.}	117.686
42	R0	{1.28208, 4.40223}	{2.07276, 11.8904}	{{1.96373, 10.9366}, {2.17882, 12.644}}	{61.6716, 170.1}	inf	0.001	76.2923	{33., 124.}	7.62913×10^{6}	2.83284	newly	0.1	38.6615	{17., 74.}	38561.5	2.52334	Reff	0.847455	0.550156	{0.392706, 0.736851}	38.1679	ar	339.	813.015	{774., 849.}	139.828
43	R0	{1.56773, 6.50474}	{2.3588, 10.0194}	{{2.32678, 9.79994}, {2.47628, 10.2848}}	{50.4596, 54.0321}	inf	12.	41.8873	{17., 72.}	250.939	0.462353	newly	11.	23.0423	{7., 44.}	125.864	0.30118	Reff	0.810516	0.361497	{0.277752, 0.463029}	55.5501	ar	498.	870.183	{834., 895.}	74.7356
44	R0	{1.10489, 6.01941}	{2.03338, 17.7256}	{{1.93882, 15.8893}, {2.12588, 19.5406}}	{84.0347, 194.474}	inf	6.	87.7344	{39., 135.}	1362.24	1.12547	newly	6.	31.4688	{13., 49.}	424.479	0.678595	Reff	0.734752	0.603896	{0.437931, 0.864937}	28.3931	ar	346.	807.344	{766., 848.}	133.336
45	R0	{1.25888, 14.1926}	{2.10481, 19.2082}	{{2.036, 18.765}, {2.1968, 19.9579}}	{67.1974, 35.3394}	inf	40.	65.3667	{29., 113.}	75.3333	0.236032	newly	16.	22.6167	{9., 40.}	65.9375	0.259708	Reff	0.723856	0.514317	{0.365651, 0.674608}	37.0351	ar	471.	821.383	{768., 858.}	74.3914
46	R0	{1.45292, 16.616}	{1.71978, 11.2412}	{{1.63746, 10.1332}, {1.81389, 12.0711}}	{18.3671, 32.3474}	inf	41.	45.9318	{15., 75.}	43.4035	0.192497	newly	19.	26.7273	{9., 42.}	61.244	0.215046	Reff	1.11003	0.713275	{0.547428, 0.959427}	35.7972	ar	494.	676.068	{607., 749.}	36.8559
47	R0	{1.22528, 16.6033}	{1.56806, 14.0552}	{{1.45954, 12.3431}, {1.73196, 19.1609}}	{27.9754, 19.5412}	inf	24.	64.1111	{21., 126.}	177.083	0.411453	newly	5.	30.5	{10., 47.}	514.444	0.758891	Reff	1.06599	1.06078	{0.761542, 1.4096}	15.9602	ar	368.	611.694	{507., 724.}	66.2364
48	R0	{1.1669, 6.92715}	{1.98149, 19.0353}	{{1.93087, 18.8819}, {1.99771, 19.6444}}	{69.8078, 174.793}	inf	8.	91.2407	{49., 141.}	1040.51	1.02279	newly	4.	32.2222	{16., 56.}	705.556	0.858029	Reff	0.768987	0.672033	{0.459547, 1.0247}	28.1221	ar	416.	790.167	{751., 836.}	89.9439
49	R0	{1.32749, 16.0887}	{1.65344, 15.2627}	{{1.55242, 12.7642}, {1.79789, 18.4377}}	{24.5542, 11.5319}	inf	24.	63.4884	{25., 113.}	168.992	0.365285	newly	26.	26.814	{10., 47.}	133.527	0.333241	Reff	1.1137	0.863541	{0.596137, 1.31083}	30.1068	ar	353.	661.465	{588., 745.}	87.3839
50	R0	{1.29636, 15.2609}	{2.02554, 17.7419}	{{1.93288, 15.9062}, {2.12588, 19.5406}}	{56.2482, 16.257}	inf	49.	88.3125	{40., 138.}	89.6684	0.259501	newly	23.	31.2656	{13., 51.}	59.4429	0.212667	Reff	0.81541	0.61427	{0.472116, 0.730924}	32.9745	ar	470.	806.328	{772., 837.}	61.5592
